# Effects of experimental N addition on plant diversity in an old‐growth temperate forest

**DOI:** 10.1002/ece3.4127

**Published:** 2018-05-02

**Authors:** Mengying Lai, Shanchuan He, Shuang Yu, Guangze Jin

**Affiliations:** ^1^ Center for Ecological Research Northeast Forestry University Harbin China; ^2^ School of Life Science and Technology Mudanjiang Normal University Mudanjiang China

**Keywords:** diversity index, interannual response, nitrogen deposition, seasonal response, soil properties

## Abstract

Temperate forest ecosystems have experienced mounting negative effects due to increasing levels of nitrogen (N) deposition. We examined the effects of experimental N addition on plant diversity in an old‐growth temperate forest to test the following hypothesis: Long‐term excessive N addition decreases plant diversity by affecting the growth of plants, which results from changes in the soil nutrient content and a decrease in the soil pH in temperate forests. Experimental N additions were administered at the following levels since 2008: control (0 kg N ha^−1 ^year^−1^), low N (30 kg N ha^−1^ year^−1^), medium N (60 kg N ha^−1 ^year^−1^), and high N (120 kg N ha^−1^ year^−1^). Additionally, plant diversity was studied from 2014 to 2016. The results showed that the experimental N additions had significant effects on plant diversity and soil properties in an old‐growth temperate forest. The high‐N treatment decreased the density, cover, and diversity of understory plants, and some herbs even appeared to undergo premature aging, whereas the species diversity of herbs and ferns in the low‐N treatment plots showed a slight increasing tendency. This may have been because the old‐growth temperate forest is an N‐limited ecosystem, so the moderate N input did not show a large influence on plant diversity. However, the long‐term high‐N treatment ultimately reduced plant diversity by changing the soil nutrient contents, decreasing the pH values, and damaging plant growth. Our results suggested that the long‐term excessive N addition negatively affected the forest ecosystem in an N‐limited temperature forest.

## 1 INTRODUCTION

Global biodiversity is decreasing at an unprecedented rate due to increased disturbances to natural environments by human activities (Sodhi, Koh, Brook, & Ng, 2004). Many factors may lead to a decrease in biologic diversity, such as habitat change, invasive species, and environmental pollution (Tilman & Lehman, 2001). Since the twentieth century, the rapid development of industry and agriculture, such as the combustion of fossil fuel, application of nitrogenous fertilizer, and rapid development of livestock production, have led to a drastic increase in the level of nitrogen (N) compounds in the atmosphere, exceeding the amounts tolerated by ecosystems (Galloway et al., 2003). Human activities have caused the average annual bulk deposition of N to increase in China from 13.2 kilograms of N per hectare in the 1980s to 21.1 kilograms of N per hectare in the 2000s (Liu et al., 2013). In addition, Zheng, Wang, Xie, Lei, and Chen (2014) found that some areas, such as the North China Plain and parts of the Northeast, reached levels of 15–30 kg N ha^−1 ^year^−1^ (Zheng et al., 2002). N compounds in the atmosphere enter ecosystems by pathways such as rainfall and strongly influence the survival of plants (Dirnbock et al., 2014), animals (Van Dobben & de Vries, 2010), and microbes (Frey, Knorr, Parrent, & Simpson, 2004). The decline in biologic diversity caused by N deposition has attracted the attention of many researchers. In fact, after land use and climate change, N deposition is considered to be the third largest factor affecting global biodiversity in the twenty‐first century (Bobbink et al., 2010).

Long‐term and site‐specific N deposition research started in the 1970s (Dirkse, Van Dobben, & Tamm, 1991), gradually forming a systematic, internationally united monitoring pattern for jointly monitoring the environmental effects of N deposition, such as water eutrophication and soil acidification (Fagerli & Aas, 2008; Sparks, Walker, Turnipseed, & Guenther, 2008). Recent studies have focused on the effects of N deposition on plant diversity in forest ecosystems (Avolio et al., 2014; Hautier, Niklaus, & Hector, 2009; Walker, Webber, Arnold, & Ebert‐May, 1994). The response of plant diversity to N deposition in forest ecosystems has received much attention because these ecosystems have the most complex community structure and the highest levels of species diversity. Although some studies have shown that forest plant diversity does not respond to N input (Davis & Thompson, 2000; Gilliam, 2006), more studies have found that N input has a negative impact on plant diversity, especially a long‐term N input. For example, after 40 years of research, McClean, Berg, Ashmore, and Preston (2011) found that N deposition, land use, and precipitation changes increased the extinction rates of native plants, and plant species in other regions of the world were also disappearing due to increasing N deposition. The mechanisms that determine the effects of N deposition on plant diversity include competition exclusion (Grime, 1973), species invasion (Bubier & Minocha, 2011), and element imbalance (Whytemare, Edmonds, Aber, & Lajtha, 1997) and can decrease plant diversity by affecting plant richness and cover. Some studies have considered that long‐term N addition may change the soil nutrient content and result in a decrease in the heterogeneity of soil nutrients, while species diversity may decrease with increases in spatial homogeneity of soil available nutrients (Gilliam, 2006; Gundale, Metlen, Fiedler, & Deluca, 2006). Others suggest that plant growth is sensitive to soil acidification, which is caused by excessive N input and damages plant roots and reduces plant diversity by affecting plant growth and nutrient balance (Roem, Klees, & Berendse, 2002; Zhao et al., 2013). Competition exclusion is considered to be the main cause of the decreasing plant diversity in temperate forests; however, recent research shows that changing soil properties related to N deposition results in more intense negative effects on plant diversity than competition exclusion (Van Den Berg et al., 2011). It is notable that previous studies only performed field surveys once per year (commonly in July), ignoring the response of the understory layer to N inputs in spring and autumn. Compared with tropical forests, northern temperate forests have clear seasons and temperature changes and the herb layer, in particular, has high richness and distinct seasonal changes (Li et al., 2008). The soil structures have different conditions in different regions of the north temperate forest, and soil nutrients, such as N significantly, change with the season (Xu, Chen, Yan, & Ji, 2008). Thus, it is necessary to study the interannual and seasonal responses of plant diversity in the understory layer of temperate forests to N addition.

As climax vegetation of northeastern China, the mixed broadleaved‐Korean pine (*Pinus koraiensis*) forest, which is characterized by a complex community structure and high diversity, occupies an important position among temperate forest types. One study found that different concentrations of N treatments had different effects on species richness in temperate mixed broadleaf conifer forests (Hu et al., 2014), in which an excessive N input decreased plant diversity by changing soil properties, specially decreasing pH values and increasing N levels (Lu, Mo, Gilliam, Zhou, & Fang, 2010). Considering the temperate forest conditions, we propose the following hypothesis: Long‐term excessive N addition decreases plant diversity by affecting the plant growth, which results from changes in the soil nutrient content and a decrease in the soil pH level in temperate forests. We studied the effects of experimental N addition on plant diversity in a typical mixed broadleaved‐Korean pine forest of northeastern China, including (1) the interannual and seasonal responses of plant diversity to N addition, (2) the effects of experimental N addition on plant density and cover, and (3) the effects of experimental N addition on soil properties that may explain the possible causes of the changes in plant diversity.

## 2 MATERIALS AND METHODS

### 2.1 Study site

Our study was conducted in the Heilongjiang Liangshui National Natural Reserve of northeastern China, at a site located on the southern slope of Xiaoxing'an mountains (47°10′50″N,128°53′20″E). This reserve is situated in Yichun City of Heilongjiang Province and is characterized by a moist temperate monsoon climate. The annual average temperature is −0.3°C, annual average precipitation is 676 mm, and precipitation from June to August accounts for over 60% of the annual precipitation. The frost‐free period is from 100 to 120 days, and the accumulated snow period is from 130 to 150 days. Natural N deposition in this reserve is 12.93 kg N ha^−1 ^year^−1^. The topography of the reserve is rolling mountainous terrain with an elevation ranging from 280 to 707 m. The forest types and plant diversity are rich with little human disturbance. This reserve is one of the largest existing preserved original Korean pine forest bases in China. The zonal soil of this area is dark brown forest soil. The main tree species is the Korean pine, accompanied by temperate deciduous tree species, such as *Fraxinus mandshurica*,* Betula costata*,* Acer tegmentosum*, and *Acer ukurunduense*.

### 2.2 Experimental treatments

N addition experimental plots were initiated in May 2008. Twelve 20 m × 20 m plots were established with each plot surrounded by a 10m‐wide buffer strip to prevent interference between groups. We performed a survey documenting the stand characteristics and soil properties before N addition and found that there was no significant variation in the stand characteristics or soil properties in the N treatment sites (Mao, Chen, & Jin, 2016). According to previous studies with similar local N deposition amounts (Lu et al., 2010; Wu et al., 2013; Zheng et al., 2014), four N addition treatments (in three replicates) were established: control (0 kg N ha^−1 ^year^−1^), low N (30 kg N ha^−1 ^year^−1^), medium N (60 kg N ha^−1 ^year^−1^), and high N (120 kg N ha^−1 ^year^−1^). Applications of a CO(NH_2_)_2_ solution were initiated in June 2008. We added N to the forest floor of each plot once per month from June to August with three equal applications. During each application, fertilizer was weighed and mixed with 20 L of water. The CO(NH_2_)_2_ solution was applied to each plot using a backpack sprayer, and control plots received 20 L of deionized water to avoid the influence of differences in the water supply.

### 2.3 Field sampling

Five 1 m × 1 m subplots were permanently established in an “X” shape in each plot in May 2014 for a total of 60 subplots. Every year, we performed a field survey of each subplot in mid‐May, mid‐July, and mid‐September to record diversity indicators, such as species, abundance, coverage, and height, of all of the vascular plants in the understory layer. To ensure that all plants were included, any individual plants that were <1 m in height were measured (Gilliam & Roberts, 2014). The cover (percentage) of the individual plant species was estimated using a square grid method. To explore the possible causes of changes in understory plant diversity, we collected soil samples in mid‐July 2016. Three soil samples were collected from 12 plots at depths of 0–10 cm. Plant litter was removed from the soil surface before samples were obtained. Fresh soil samples were passed through a 2‐mm sieve for further analysis after stones, and thick roots were removed by hand. The soil pH was measured by a laboratory pH meter, and the soil total N, total phosphorus (P), and available P were measured using a 722N visible spectrophotometer (325–1,100 nm) (Shanghai Precision &Scientific Instrument Co., Ltd., China) as well as Mo‐Sb colorimetry methods. Total N was measured using a TM 2300 auto Kjeldahl analyzer (Foss Teactor AB, Hoganas, Sweden).

### 2.4 Statistical analysis

All of the understory plants in this study were classified into different functional groups: tree seedlings, shrubs, herbaceous, and ferns (Gilliam & Roberts, 2014). Ferns were included because of their unique reproductive system and sensitivity to environmental changes. To simultaneously test for overall N treatment effects over time for the study period from 2014 to 2016, we used three‐way repeated‐measures analysis of variance (ANOVA) with Tukey's honestly significant difference (HSD) test, which analyzed the effects of N treatment, year, and season on understory plants. To test the seasonal response of species diversity to N addition for different functional groups, we used one‐way ANOVA with Tukey's HSD test, which analyzed the differences in density and cover between the four treatments in the growing season in 2016. We also conducted contrast analysis to test the differences in richness (mean number of species per m^2^ in each replication), stem density (mean number of plants per m^2^ in each replication), cover (mean percentage cover of plants in each replication), and their relative value for different years for the same treatment. Relative values were calculated as the plot average for the specified period divided by the average diversity of species in the control plot over the same period. In addition, we also analyzed the response of the α‐diversity index of the herbaceous community (the herb group has enough numbers of species and individuals) to N addition for different years. The Vegan package in the software program R was used to calculate the herbaceous α‐diversity, including the Shannon–Wiener index, Simpson index, and Pielou index. ANOVA and Tukey's HSD test were conducted using SPSS 22.0 for Windows, and Origin8.0 was used to plot the data.

## 3 RESULTS

Sampling within our plots produced a total of 49, 61, and 45 species belonging to four functional groups from three seasons (i.e., spring, summer, and autumn) in 2016. Among the four functional groups, herbs were dominant in species richness, density, and cover, accounting for 60% of the total species number in all plots. Herb species had the highest richness level in summer. The species numbers of tree seedlings and shrubs in the treatment site were lower than those in the control site for three seasons. The average height of tree seedlings was 10.52 cm, and almost all were newborn seedlings that were sensitive to N addition. Ferns never appeared in the high‐N plots over the whole year, and the species numbers of ferns in the low‐N and medium‐N plots were greater than those in the control plots (Appendix S1). The effect of N addition on the species average height varied for different species. The average height of most species in the control plots was greater than that in the high‐N plots, but lower than that in low‐N and medium‐N plots (Appendix S2). Compared to those in the control plots, the soil properties showed significant variations in the high‐N plots in July 2016. The total N and available P in soil were greater with elevated N treatment levels, and the values in the high‐N plots were significantly higher than those in the control plots. By contrast, the soil pH decreased with increasing levels of N addition (Table 1).

**Table 1:** Response of soil properties to N addition for a typical mixed broadleaved‐Korean pine forest in Xiaoxing'an mountains

Treatment	Soil pH	Total N mg/g	Total P mg/g	Available P mg/kg
Control	5.64 (0.07)a	8.70 (1.07)b	0.98 (0.09)a	11.97 (1.65)b
Low N	5.35 (0.12)ab	10.07 (1.17)a	1.05 (0.15)a	20.65 (4.15)ab
Medium N	5.06 (0.21)ab	10.76 (1.23)ab	0.99 (0.10)a	20.40 (3.94)ab
High N	5.00 (0.08)b	13.93 (1.40)a	1.25 (0.14)a	21.06 (2.04)a

Values are the means ± *SE*. Values with different letters are significantly different (*p *< .05).

### 3.1 Interannual response of the species richness of functional groups to N addition

Repeated‐measures ANOVA showed significant effects of N treatment, year, and season on the species richness of tree seedlings and herbaceous plants, and there was a significant interaction between N treatment and season for ferns. In addition, season did not significantly affect the richness of shrubs, and year did not significantly affect the richness of ferns. However, no groups responded significantly to the interaction of all three factors (Table 2).

**Table 2:** Response of species richness to N addition, year, and season of different functional groups in three seasons. The *F*‐values and *p*‐values in parentheses are shown, bold values denote significant effects(p<0.05)

Effect	*df*	Tree seedlings	Shrubs	Herbaceous	Ferns
Nitrogen	3	**19.15 (<0.001)**	**36.53 (<0.001)**	**13.28 (<0.001)**	**35.77 (<0.001)**
Year	2	**8.25 (0.001)**	**7.66 (0.001)**	**14.25 (<0.001)**	3.05 (0.054)
Season	2	**10.62 (<0.001)**	2.21 (0.117)	**80.41 (<0.001)**	**15.13 (<0.001)**
N×Y	6	2.21 (0.052)	0.58 (0.749)	0.21 (0.974)	1.16 (0.338)
N×S	6	1.70 (0.134)	0.76 (0.602)	1.31 (0.266)	**3.84 (0.002)**
Y×S	4	**2.54 (0.047)**	1.45 (0.227)	1.73 (0.152)	**4.27 (0.004)**
N×Y×S	12	1.06 (0.410)	0.46 (0.931)	0.56 (0.870)	0.57 (0.857)

**Figure 1:**
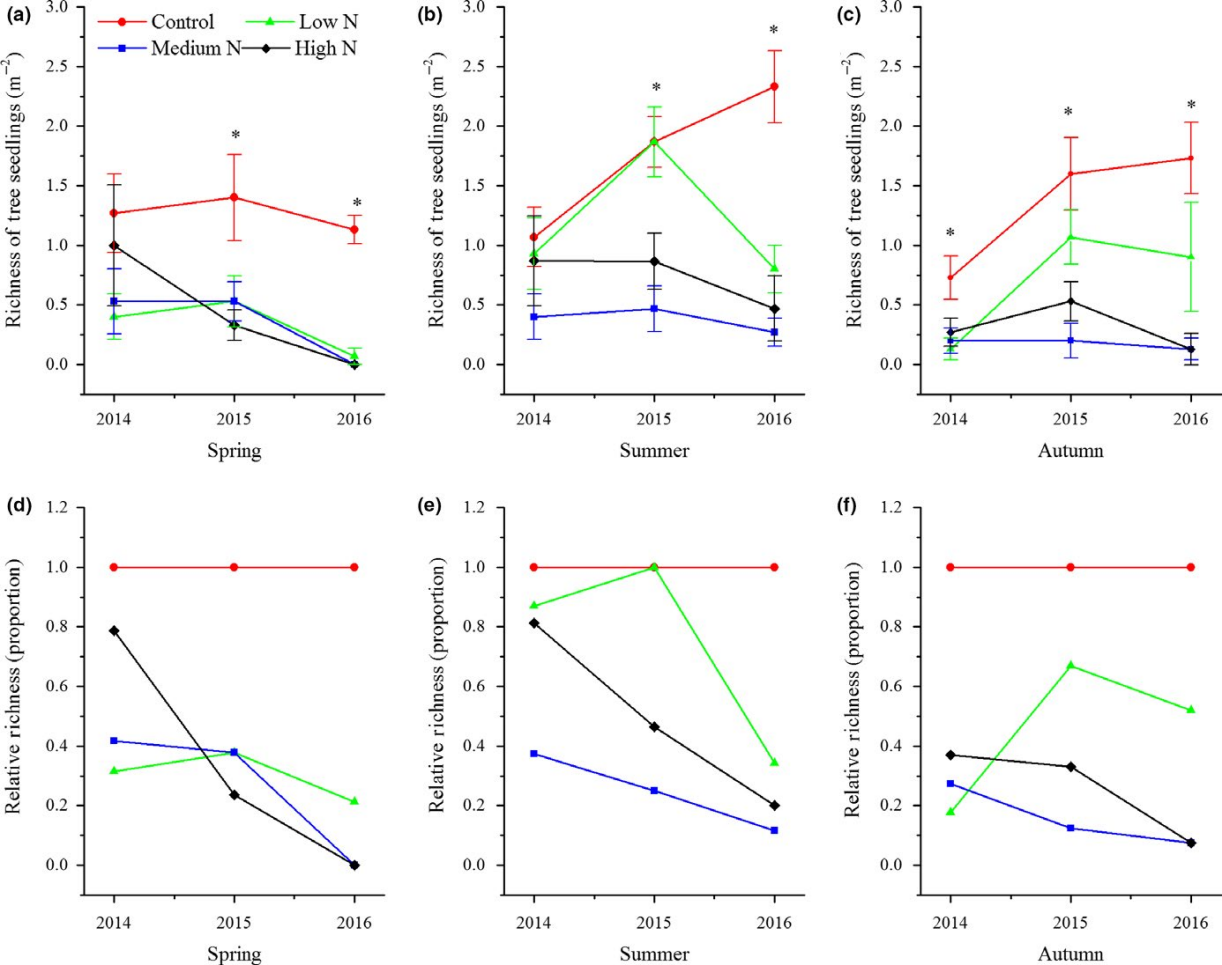
Interannual response of species richness of tree seedlings to N addition for the years 2014–2016. Values are the means ± *SE* (a–c) richness of tree seedlings in three seasons; (d–f) relative richness of tree seedlings of a specific season. *Significant difference between control plots against N treatment plots

**Figure 2:**
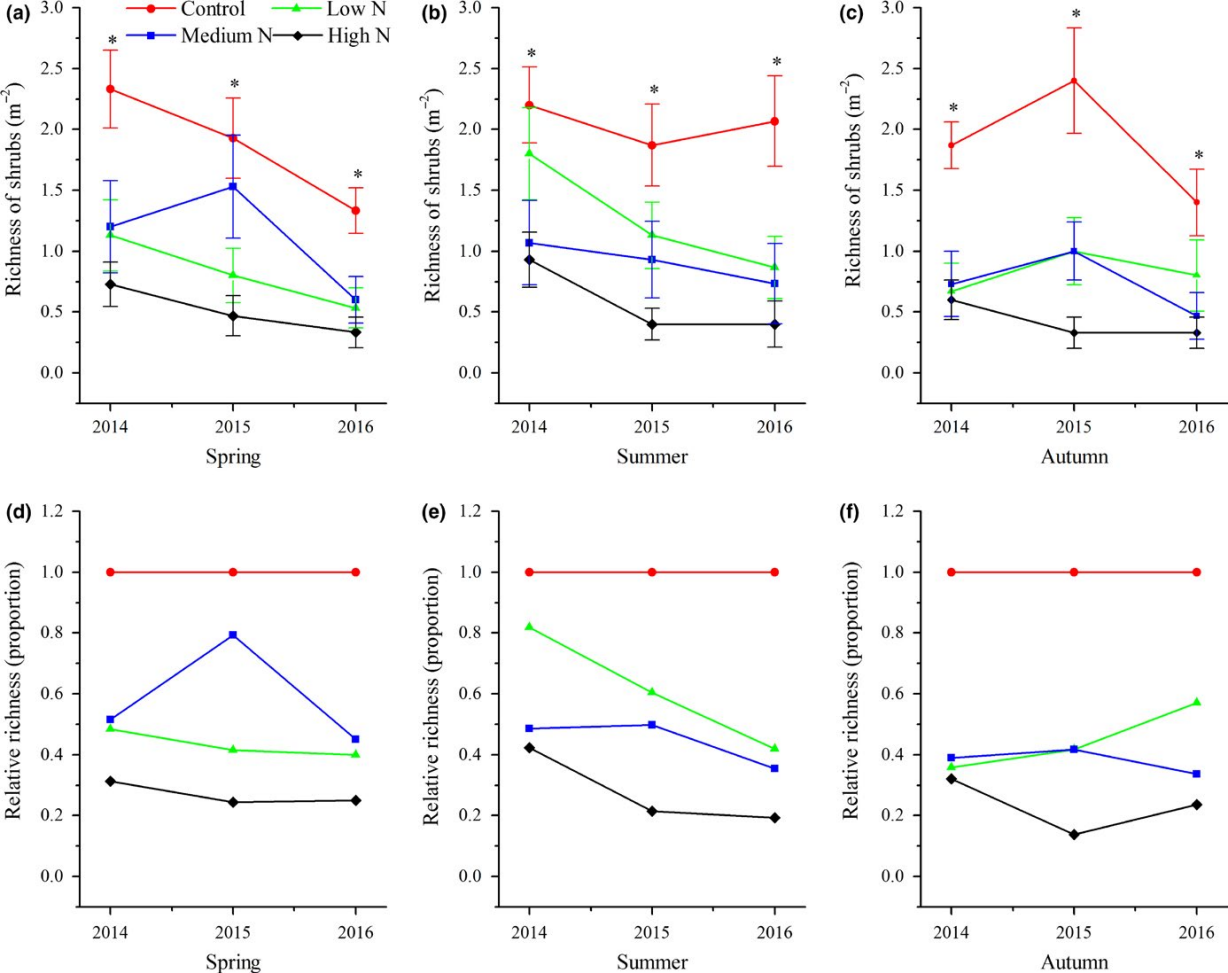
Interannual response of species richness of shrubs to N addition for the years 2014–2016. Values are the means ± *SE*; (a–c) richness of shrubs in three seasons; (d–f) relative richness of shrubs of a specific season. *Significant difference between control plots against N treatment plots

**Figure 3:**
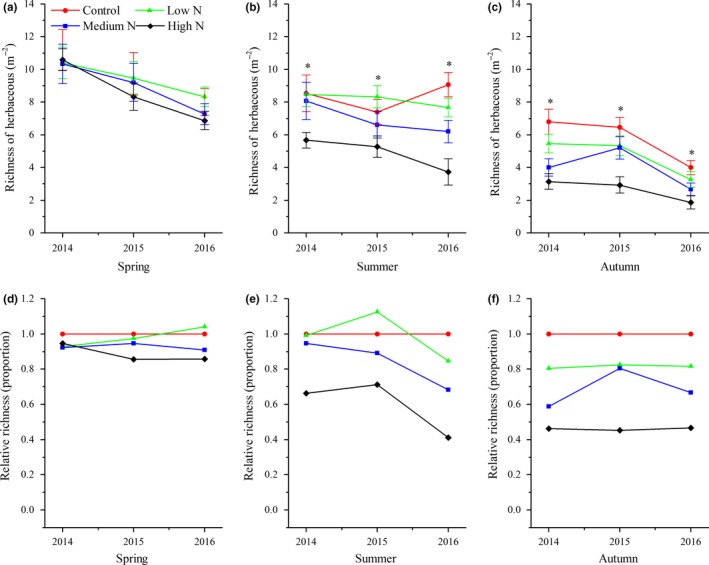
Interannual response of species richness of herbs to N addition for the years 2014–2016. Values are the means ± *SE*; (a–c) richness of herbs in three seasons; (d–f) relative richness of herbs of a specific season. *Significant difference between control plots against N treatment plots

**Figure 4:**
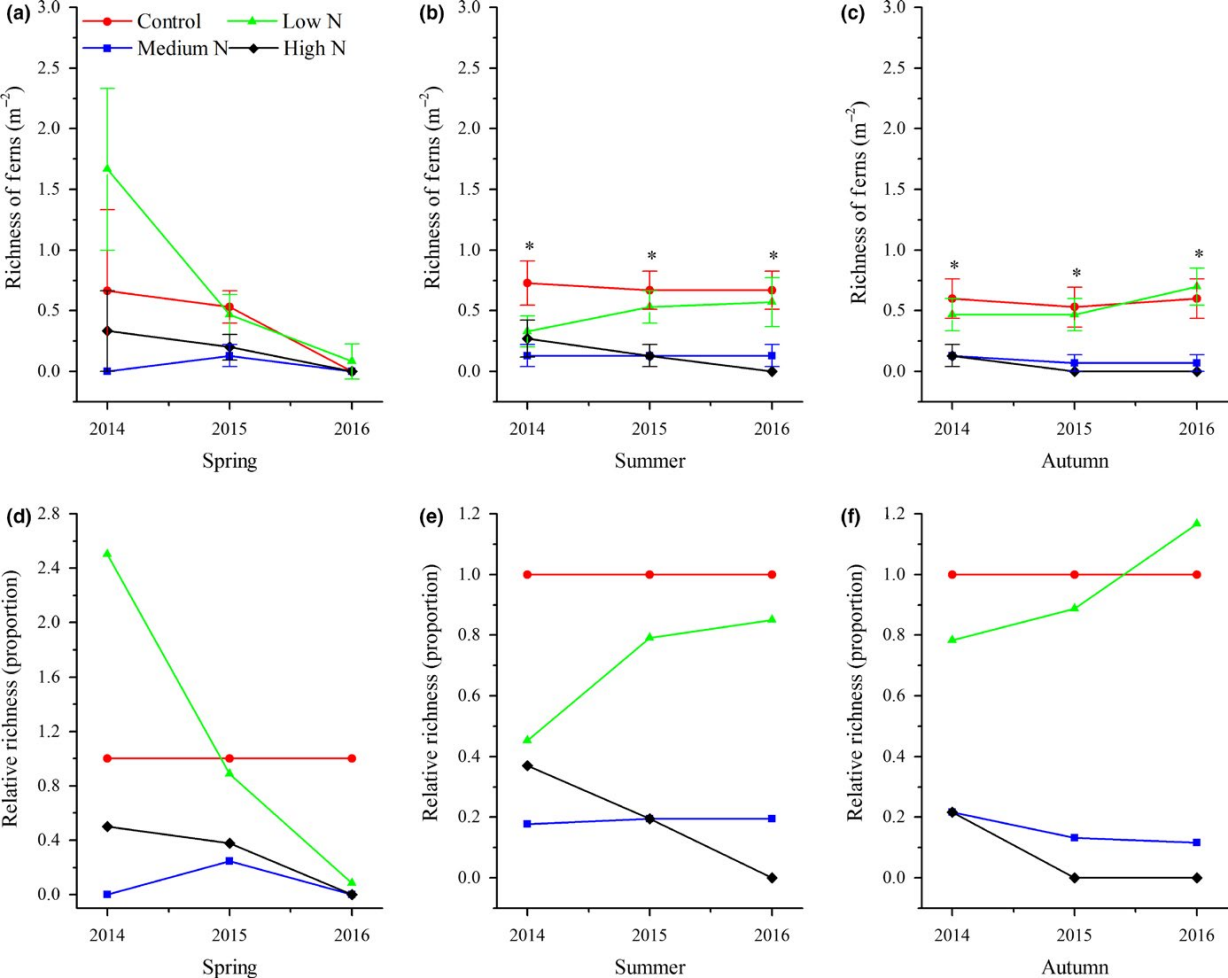
Interannual response of species richness of ferns to N addition for the years 2014–2016. Values are the means ± *SE*; (a–c) richness of ferns in three seasons; (d–f) relative richness of ferns of a specific season. *Significant difference between control plots against N treatment plots

During the study period, tree seedling richness in spring and summer began to show significant variations after 2015 (Figure 1a,b), while significant changes were noted between control and treatment plots in autumn (Figure 1c). In the high‐N plots, tree seedling richness decreased most rapidly in spring, decreasing by approximately 80%, and decreased by 60%–90% in autumn from 2014 to 2016. In the low‐N plots, tree seedling richness showed minor variations across three years (Figure 1d–f). Compared with the control plots, the effects of N addition on plant richness of shrubs in the high‐N plots across three seasons started in 2014 (Figure 2d–f). There were no significant variations in herbaceous richness between the control and treatment plots in spring for all three years, while the opposite results were observed in summer and autumn (Figure 3a,b). The relative richness of herbs showed almost no change between the control and treatment plots in spring (Figure 3d). The decrease of herbaceous richness in the high‐N plots in the summer of 2016 was more than that noted during the last two years (Figure 3e). Ferns are sensitive to N input, and the richness of ferns in the low‐N plots in summer and autumn showed a slight increasing trend from 2014 to 2016, as well as a continuously decreasing trend in the same period for the high‐N plots. In 2016, ferns were never found in the high N plots (Figure 4e,f).

### 3.2 Seasonal response of the density and cover of functional groups to N addition

**Figure 5:**
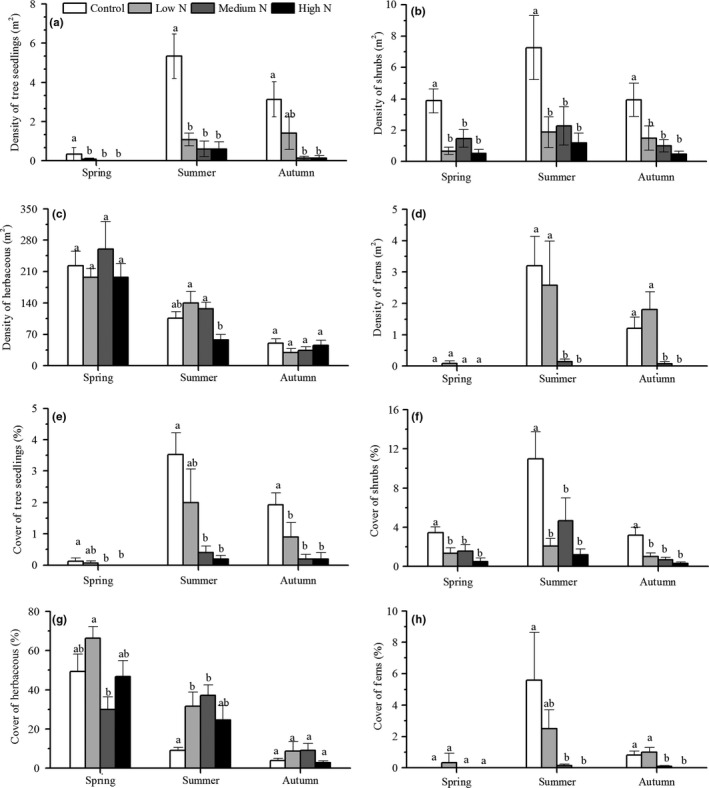
Seasonal response of the density and cover of functional groups to N addition in 2016. Values are the means ± *SE*. Values with different letters are significantly different (*p* < .05)

The density and cover of herbs showed a decreasing tendency over time from spring to autumn in 2016, while others showed an initial increasing trend followed by a decreasing trend (Figure 5a–d). Herbs only showed significant variations in the summer, with the density in the low‐N plots and medium‐N plots being significantly greater than that in the high‐N plots (Figure 5c). The shrub density and cover in all of the treatment plots were significantly less than those in the control plots during all three seasons (Figure 5b–f). Tree seedlings showed similar trends as shrubs, except for the density in low‐N plots in autumn (Figure 5a) and cover in spring and summer (Figure 5e). Ferns in the low‐N plots appeared earlier than those in the control plots, while medium and high‐N additions significantly decreased the densities in all three seasons (Figure 5d). No significant differences were noted in fern cover among treatments in spring, and the cover of the medium‐N and high‐N plots was below that of the control plots (Figure 5h).

### 3.3 Seasonal response of the herbaceous diversity and average height to N addition in 2016

Our study found that the herbaceous community was the dominant community of the understory layer, accounting for 70% of the community. The dominant species of the herb layer was *Carex pilosa*.

Significant responses of the herbaceous community diversity of the mixed broadleaved‐Korean pine forest were noted in response to N addition over time (Table 2). N input showed no obvious effects on the indices of plant diversity in 2014 and 2015, but a high N addition decreased the Shannon–Wiener index, Simpson index, and Pielou index in 2016. During our study period, only diversity indices in the high‐N plots were found to be significantly decreased compared to those of the other treatments. The species number and average height of most herbs in the high‐N plots were lower than those in the control plots throughout the whole year. Some species never appeared in the high‐N treatment plots, such as *Oxalis corniculata* and *Urtica angustifolia*. In addition, moderate N additions increased the numbers of many species, and the numbers of many species in the low‐N plots were exceeded by those in the control plots. The number of dominant species (i.e., *Carex pilosa*) in all of the treatment plots exceeded that in the control plots, and some species, such as *Maianthemum bifolium*, even showed early senescence and death (Appendices S1 and S2).

## 4 DISCUSSION

### 4.1 Effect of experimental N addition on understory plant diversity in a typical mixed broadleaved‐Korean pine forest

We demonstrated that N addition significantly decreased understory plant diversity in a typical mixed broadleaved‐Korean pine forest, especially in the high‐N plots (Figures 1, 2, 3, 4). This result is similar to those reported by other studies on temperate forests (Gilliam, 2006; Rainey, Nadelhoffer, Silver, & Downs, 1999; Strengbom, Nordin, Nasholm, & Ericson, 2002). Competition is considered to be the main reason for the decline of diversity in temperate forests (Gilliam, 2006). Bobbink et al. (2010) found that N deposition greatly threatens plant diversity in temperate and northern parts of North America and Europe as N deposition is a major driving factor for species composition change. Species that cannot adapt to the environment disappear due to competitive exclusion, eventually leading to lower diversity levels. For example, McClean et al. (2011) studied N deposition in Europe from the 1950s to the 1990s and found that environmental factors, such as N deposition and land use, significantly reduced diversity by driving local plant extinctions and changing plant competitiveness. In addition, recent research has also found that there are negative correlations between soil acidification and plant diversity, specially that continuous input of N causes soil acidification, reduces base cations (e.g., Ca^2+^ and Mg^2+^), damages plant roots, affects nutrient balance and normal growth, and ultimately decreases diversity (Lu, Mao, Gilliam, Luo, & Mo, 2014; Roem et al., 2002; Rowe et al., 2006). Van Den Berg et al. (2011) also found that both soil acidification caused by N deposition and N input had negative effects on the number of plant species. Our findings support this conclusion. North temperate forests are generally considered to be N‐limited ecosystems, and most plant species from such ecosystems are adapted to low‐N conditions. Long‐term N input consistently increases the soil N content of N‐limited forests and affects the growth of some species, and these species (nitrophytes) have an improved competitive advantage and exclude other species, resulting in an ultimate decrease of overall diversity (Grime, 1973). We also found that the sensitivities of different groups responding to N additions were different. Our results indicated that the richness of tree seedlings and ferns was reduced to a greater extent under the high‐N treatment. This result was similar to that of Lu et al. (2010), who studied the effects of N addition on plant diversity in the Dinghushan Biosphere Reserve, an N‐saturated forest, and who also found that reductions of tree seedlings and ferns corresponded to N‐mediated declines. It is notable that in their study, the dominant species was tree seedlings and that N addition did not lead to an increase in any plant growth. These results significantly differ from those of this study. Their study site was located in an N‐saturated forest, and the input of N deposition decreased plant diversity due to N‐mediated changes. However, our study site was located in an N‐limited forest, in which moderate N addition not only increased the number of some nitrophilous species but also promoted growth.

In addition, our research found that there were significant responses in the richness of four functional groups to season, while different functional groups varied differently with season (Figure 5). The density and cover of ferns and herbs showed no significant decrease with N addition in spring, but tree seedlings and shrubs showed significant decreases among three seasons (Figure 5). Many studies have found that N input significantly changes the element contents and biomass of woody seedling leaves, roots, and stems; affects photosynthesis; and decreases the root:shoot ratio and plant diversity (Li et al., 2005; Roem & Berendse, 2000; Whytemare et al., 1997). Although tree seedlings and shrubs are perennial plants, the species of these groups in our study site were seedlings and were more sensitive to environmental stress. Hence, we hypothesize that long‐term N addition in our site changed the soil nutrient content, created a nutrient imbalance of woody plant seedlings, caused increased levels of seedling mortality, and, ultimately, led to lower diversity. On the other hand, most herbs are therophytes, which are abundant species with obvious seasonal dynamics and early‐spring herbs. Simultaneously, many studies have shown that the effect of N deposition on colonized and lost species is a long process (Isbell, Tilman, Polasky, Binder, & Hawthorne, 2013; Keith, Newton, Morecroft, Bealey, & Bullock, 2009; McClean et al., 2011). We believe that the period of N addition in this study site was not long enough. Herbaceous species richness in spring increased because of the appearance of early‐spring plants, while N deposition did not show an obvious inhibiting effect on the appearance of early‐spring plants, so there was no significant variation in plant species richness in spring among treatments and there was a slight decreasing tendency over the years. Finally, excess N input showed apparently negative effects on ferns, which are sensitive to environmental changes because of their unique reproductive methods (Coomes et al., 2005; George & Bazzaz, 2014). In our study, we obtained similar results, but we also found that a moderate N input was conducive to the growth of ferns. This may be because our study site was located in an N‐limited temperate forest in which a moderate N addition can increase the productivity of ferns.

### 4.2 The effects of experimental N addition on herbaceous species diversity in a typical mixed broadleaved‐Korean pine forest

As the dominant community of our site, we found that there were significant responses of herbaceous community diversity (Simpson index, Shannon–Wiener index, and Pielou index) to N addition over time in a mixed broadleaved‐Korean pine forest (Table 3) and that a moderate N input increased the density and cover of herbaceous plants in summer (Figure 5c,g). After 9 years of N addition, the herbaceous community diversity index showed a significant decrease in 2016 (Table 3). Apparently, the long‐term excess N input not only decreased species richness but also decreased species abundance and evenness, while a moderate N input increased diversity. Cleland and Harpole (2010) obtained similar results and showed that N fertilizer obviously reduced the α‐diversity. In the *Pinus tabulaeformis* of Taiyue Mountain, Li et al. (2015) found that N addition decreased the α‐diversity in natural forests. The decrease of the herbaceous community diversity was often thought to be related to the N availability of herbs. Eutrophic species with rapid N transformation rates prefer to exist in N‐rich conditions, while oligotrophic species with slow N transformation rates disappear gradually (Aerts & Iii, 2000; Aerts & Peijl, 1993) and plant diversity ultimately decreases. Dirnbock et al. (2014) studied data from 1335 permanent plots of 28 forests in Europe and found that the eutrophication of ecosystems, which was caused by long‐term N deposition, was the main reason for the threatened plant diversity. Different forest ecosystems have their own critical loads, and N addition does not produce negative effects on ecosystems when the level of N addition is below the critical load and vice versa. Our study site is located in a N‐limited temperate forest in which plants are adapted to N‐limited conditions, causing the long‐term high‐N‐input treatment to reduce the α‐diversity by affecting the growth of plants, resulting in a change in nutrient conditions. However, the low‐N and medium‐N treatments did not show significant effects on plant diversity.

**Table 3:** Response of the herbaceous community diversity indices to N addition in summers of different years of a typical mixed broadleaved‐Korean pine forest in Xiaoxing'an mountains

Years	Treatment	Simpson index	Shannon–Wiener index	Pielou index
2014	Control	0.63 (0.03)Aa	1.31 (0.10)Aa	0.71 (0.04)Aa
	Low N	0.60 (0.04)Aa	1.32 (0.11)Aa	0.66 (0.05)Aa
	Medium N	0.49 (0.07)Aa	1.04 (0.16)Aa	0.50 (0.07)Aa
	High N	0.49 (0.06)Aa	0.97 (0.12)Aa	0.57 (0.06)Aa
2015	Control	0.68 (0.03)Aa	1.45 (0.08)Aa	0.75 (0.04)Aa
	Low N	0.64 (0.05)Aa	1.41 (0.12)Aa	0.68 (0.05)Aa
	Medium N	0.51 (0.07)Aa	1.08 (0.15)Aa	0.57 (0.05)Aa
	High N	0.41 (0.05)Aa	0.86 (0.13)Aa	0.54 (0.05)Aa
2016	Control	0.64 (0.04)Aa	1.46 (0.11)Aa	0.67 (0.03)Aa
	Low N	0.66 (0.04)Aa	1.40 (0.10)Aa	0.70 (0.04)Aa
	Medium N	0.47 (0.08)Ab	1.02 (0.17)Aa	0.53 (0.07)Aab
	High N	0.32 (0.07)Ab	0.62 (0.15)Ab	0.46 (0.09)Ab

Values are the means ± *SE*. Values with different capital letters are significantly different within years; small letters are significantly different within treatments (*p *< .05).

It is notable that the high‐N treatment decreased the species number and average height of most herbaceous plants and that some species never appeared in the high‐N plots. However, the species number and height of some herbaceous plants were increased in response to a moderate N input. We also observed that some species appeared early senescence and death (Appendices S1 and S2). We suggest that excessive N addition decreased the herb plant diversity by affecting the growth rate of plants by changing the availability of plant nutrients, resulting from altered soil nutrients (especially N and P). Gilliam et al. (2016) found that N addition simultaneously increased the number of nitrophilous species and decreased the number of N‐efficient species. Excess N input increased the species homogeneity and led to low plant diversity. On the other hand, N and P are related to plant growth in most ecosystems. For example, Fraterrigo, Turner, and Pearson (2006) found that the herbaceous growth rates were correlated with N and P levels in southern Appalachian forests. Agren, Wetterstedt, and Billberger (2012) found that N and P were two elements that simultaneously limited the plant growth and that any increase in either increased the plant growth rate. Thus, we suggest that a moderate N input increases the number of nitrophilous species and promotes plant growth, as reflected by plant height.

## 5 CONCLUSION

Significant effects of experimental N additions on plant diversity and soil properties were noted in an old‐growth temperate forest. Long‐term excessive N addition decreased plant diversity by affecting the growth of plants due to changes in the soil nutrient content and decreases in the soil pH in temperate forests. The high‐N treatment decreased species richness, density, and cover, whereas the low‐N treatment showed slightly positive effects on the species richness of herbs and ferns. The number of some species increased in response to the moderate N addition. The moderate N addition promoted the growth of some nitrophilous species and had a positive influence on plant diversity, while the long‐term high‐N treatment changed the soil characteristics, decreased the soil pH values, damaged plant growth, and ultimately reduced plant diversity. Lastly, some herbaceous species appeared to undergo premature aging and death, which may have resulted from the increased growth rate caused by the changed soil properties, such as the N:P ratio, but the exact mechanism determining how N addition affects plant growth rates and what species are most easily affected warrants further research.

## AUTHOR CONTRIBUTION

MYL and GZJ conceived the idea. MYL and SCH collected the plant diversity data. MYL analyzed the data. MYL and GZJ wrote the manuscript; other authors provided editorial advice.
