# Supplementary material for: Effects of experimental N addition on plant diversity in an old‐growth temperate forest
Source: Ecol Evol. 2018 May 2;8(11):5900–11. doi: 10.1002/ece3.4127 (PMC6010721; doi:10.1002/ece3.4127)
Supplement: Supplementary file 1 [file ECE3-8-5900-s001.docx]

**Appendix**

**Appendix S1** Seasonal changes in the plant species number (per 15 m^2^) in a mixed typical mixed broadleaved-Korean pine forest in Xiaoxing’an Mountains in 2016

**Appendix S2** Seasonal changes in the plant species average height (cm) in a mixed typical mixed broadleaved-Korean pine forest in Xiaoxing’an Mountains in 2016

**Appendix S1**

| Functional  groups | Species | Family | Spring | | | |  | Summer | | | |  | Autumn | | | |
| --- | --- | --- | --- | --- | --- | --- | --- | --- | --- | --- | --- | --- | --- | --- | --- | --- |
|  |  |  | N1 | N2 | N3 | N4 |  | N1 | N2 | N3 | N4 |  | N1 | N2 | N3 | N4 |
| tree seedlings | *Acer tegmentosum* | Aceraceae | 2 | - | - | - |  | 17 | 1 | 1 | 2 |  | 7 | - | - | 1 |
| tree seedlings | *Fraxinus mandshurica* | Oleaceae | 1 | - | - | - |  | 35 | 6 | - | 5 |  | 21 | 3 | - | - |
| tree seedlings | *Ulmus japonica* | Ulmaceae | 6 | - | - | - |  | 1 | - | - | - |  | - | - | - | - |
| tree seedlings | *Ulmus laciniata* | Ulmaceae | 1 | 1 | - | - |  | 3 | 1 | - | 2 |  | 2 | 3 | - | - |
| tree seedlings | *Corylus mandshurica* | Betulaceae | - | - | - | - |  | 11 | 1 | - | - |  | 9 | 1 | 1 | - |
| tree seedlings | *Acer mono* | Aceraceae | 6 | - | - | - |  | 4 | - | - | - |  | 2 | - | - | 1 |
| tree seedlings | *Abies nephrolepis* | Pinaceae | - | - | - | - |  | 4 | 1 | - | - |  | 2 | 2 | - | - |
| tree seedlings | *Pinus koraiensis* | Pinaceae | - | - | - | - |  | 3 | 4 | 8 | - |  | 2 | 5 | 1 | - |
| tree seedlings | *Acer ukurunduense* | Aceraceae | 2 | - | - | - |  | 3 | 2 | - | - |  | - | - | - | - |
| tree seedlings | *Prunus padus* | Rosaceae | - | - | - | - |  | - | 1 | - | - |  | - | - | - | - |
| shrubs | *Acanthopanax senticosus* | Araliaceae | 13 | 1 | 1 | 4 |  | 11 | 2 | - | 5 |  | 9 | 1 | 1 | 3 |
| shrubs | *Lonicera chrysantha* | Caprifoliaceae | 27 | - | - | - |  | 47 | 3 | 6 | - |  | 31 | 2 | - | - |
| shrubs | *Philadelphus schrenkii* | Hydrangeaceae | 1 | - | - | - |  | 13 | 1 | 1 | 2 |  | 1 | - | - | - |
| shrubs | *Euonymus pauciflorus* | Celastraceae | 9 | 3 | 1 | 3 |  | 10 | 13 | 11 | 6 |  | 8 | 8 | 7 | 2 |
| shrubs | *Deutzia glabrata* | Hydrangeaceae | 7 | 6 | 5 | 1 |  | 16 | 6 | 1 | 5 |  | 8 | 2 | - | 2 |
| shrubs | *Sorbaria sorbifolia* | Rosaceae | 1 | - | 3 | - |  | - | - | - | - |  | - | - | - | - |
| shrubs | *Ribes mandshuricum* | Grossulariaceae | - | - | 7 | - |  | 1 | - | 9 | - |  | 1 | - | 4 | - |
| shrubs | *Spiraea salicifolia* | Rosaceae | - | - | 5 | - |  | - | - | 4 | - |  | - | - | 3 | - |
| shrubs | *Syringa reticulata* var.*amurensis* | Oleaceae | - | - | - | - |  | 3 | 2 | - | - |  | 1 | 1 | - | - |
| shrubs | *Actinidia kolomikta* | Actinidiaceae | - | - | - | - |  | 7 | - | 2 | - |  | - | - | - | - |
| shrubs | *Berberis poiretii* | Berberidaceae | - | - | - | - |  | 1 | - | - | - |  | - | - | - | - |
| shrubs | *Vitis amurensis* | Vitaceae | - | - | - | - |  | - | 1 | - | - |  | - | 1 | - | - |
| Herbaceous | *Cardamine macrophylla* | Cruciferae | 1 | 2 | - | - |  | 8 | 9 | 16 | 9 |  | 18 | 5 | 21 | 5 |
| Herbaceous | *Lamium barbatum* | Labiatae | 2 | 2 | - | 3 |  | 5 | 11 | 3 | - |  | - | - | - |  |
| Herbaceous | *Paris verticillata* | Liliaceae | 2 | 45 | 5 | 19 |  | 4 | 1 | - | - |  | - | - | - | - |
| Herbaceous | *Carex pilosa* | Cyperaceae | 640 | 263 | 1302 | 1033 |  | 182 | 359 | 731 | 297 |  | 65 | 146 | 178 | 130 |
| Herbaceous | *Dichocarpum sutchuenense* | Ranunculaceae | 108 | 39 | 84 | 30 |  | 34 | 23 | 19 | 25 |  | - | - | 1 |  |
| Herbaceous | *Bupleurum sibiricum* | Umbelliferae |  | - | - | - |  | 42 | - | - | - |  | 1 | - | - | - |
| Herbaceous | *Heracleum hemsleyanum* | Umbelliferae | - | - | - | - |  | - | - | - | 1 |  | - | - | - | - |
| Herbaceous | *Parasenecio auriculatus* | Compositae | 34 | 4 | 39 | - |  | 2 | 39 | 18 | - |  | 1 | - | - | - |
| Herbaceous | *Adenocaulon himalaicum* | Compositae | - | - | - | - |  | - | - | - | - |  | - | - | - | 1 |
| Herbaceous | *Hylomecon japonica* | Papaveraceae | 660 | 335 | 128 | 228 |  | 17 | 13 | 6 | 10 |  | - | - | - | - |
| Herbaceous | *Polemonium caeruleum* | Polemoniaceae | - | - | 1 | - |  | 3 | - | - | - |  | - | - | - | - |
| Herbaceous | *Scutellaria pekinensis* | Labiatae | - | - | - | - |  | 88 | 92 | 2 | - |  | 10 | 3 | - | - |
| Herbaceous | *Theligonum macranthum* | Theligonaceae | 12 | 9 | 17 | 2 |  | 8 | 19 | 5 | 6 |  | 1 | - | - | - |
| Herbaceous | *Aruncus sylvester* | Rosaceae | - | - | - | 2 |  | - | - | - | - |  | - | - | - | - |
| Herbaceous | *Chrysosplenium pseudofauriei* | Saxifragaceae | 436 | 262 | 320 | 2 |  | 163 | 619 | 287 | 30 |  | 19 | 192 | 123 | - |
| Herbaceous | *Carex siderosticta* | Cyperaceae | - | - | - | - |  | 32 | - | 2 | - |  | - | - | - | 25 |
| Herbaceous | *Urtica laetevirens* | Urticaceae | - | - | 41 | - |  | 2 | 6 | - | - |  | 25 | 6 | - | - |
| Herbaceous | *Heracleum barbatum* | Umbelliferae | 4 | 3 | 3 | 14 |  | 4 | - | 2 | - |  | - | - | - | - |
| Herbaceous | *Veratrum dahuricum* | Liliaceae | - | - | 6 | - |  | - | - | 1 | - |  | - | - | - | - |
| Herbaceous | *Rubia sylvatica* | Rubiaceae | 5 | - | 72 | 12 |  | 27 | 104 | 52 | 38 |  | - | - | - | 2 |
| Herbaceous | *Diarrhena manshurica* | Gramineae | - | - | - | - |  | 1 | - | - | 13 |  | - | 1 | 32 | - |
| Herbaceous | *Caltha palustris* | Ranunculaceae | 13 | - | 12 | 4 |  | - | 22 | 10 | - |  | 129 | - | 7 | - |
| Herbaceous | *Equisetum hyemale* | Equisetaceae | 109 | - | - | - |  | 137 | - | - | - |  | - | - | - | - |
| Herbaceous | *Viola collina* | Violaceae | - | - | - | - |  | 34 | 1 | - | - |  | 6 | 1 | 4 | - |
| Herbaceous | *Brachybotrys paridiformis* | Boraginaceae | 3 | - | - | - |  | - | - | 12 | - |  | - | - | - | - |
| Herbaceous | *Circaea alpina* | Onagraceae | - | - | - | - |  | - | - | 11 | - |  | - | - | - | - |
| Herbaceous | *Impatiens nolitangere* | Balsaminaceae | 54 | 24 | 352 | 264 |  | 4 | 91 | 240 | 159 |  | - | 5 | - | - |
| Herbaceous | *Thalictrum przewalskii* | Ranunculaceae | - | - | - | - |  | - | - | 22 | - |  | - | - | - | - |
| Herbaceous | *Phryma leptostachya* subsp*. asiatica* | Labiatae | - | - | - | - |  | 3 | - | - | - |  | 1 | 1 | - | - |
| Herbaceous | *Pilea pumila* | Urticaceae | - | - | - | - |  | - | - | - | - |  | - | 26 | - | - |
| Herbaceous | *Eranthis stellata* | Ranunculaceae | 30 | 22 | 11 | 16 |  | - | - | - | - |  | - | - | - | - |
| Herbaceous | *Carex ussuriensis* | Cyperaceae | - | - | 34 | 11 |  | 1 | - | 4 | - |  | - | - | - | - |
| Herbaceous | *Aconitum volubile* | Ranunculaceae | 1 | 23 | 18 | 20 |  | - | 8 | 6 | 3 |  | - | - | - | - |
| Herbaceous | *Adoxa moschatellina* | Adoxaceae | 213 | 82 | 369 | 348 |  | - | 7 | 7 | - |  | - | - | - | - |
| Herbaceous | *Maianthemum bifolium* | Liliaceae | 35 | 10 | 21 | 2 |  | 33 | 21 | 8 | 1 |  | 1 | - | - | - |
| Herbaceous | *Urtica angustifolia* | Urticaceae | 6 | 5 | - | - |  | 1 | 11 | 36 | - |  | - | 10 | 19 | - |
| Herbaceous | *Aegopodium alpestre* | Umbelliferae | 229 | 40 | 224 | 93 |  | 285 | 244 | 273 | 239 |  | 113 | 175 | 168 | 131 |
| Herbaceous | *Corydalis yanhusuo* | Fumariaceae | 181 | 179 | 150 | 273 |  | - | - | - | - |  | - | - | - | - |
| Herbaceous | *Saussurea amurensis* | Compositae | 1 | - | 2 | 4 |  | - | - | 3 | 2 |  | - | - | - | 1 |
| Herbaceous | *Carex rigescens* | Cyperaceae | 2 | - | 18 | 1 |  | 48 | 4 | 36 | 1 |  | 23 | 2 | 13 | 3 |
| Herbaceous | *Anemone cathayensis* | Ranunculaceae | 1230 | 388 | 550 | 706 |  | - | - | - | - |  | - | - | - | - |
| Herbaceous | *Filipendula palmata* var. *glabra* | Rosaceae | 111 | 19 | 119 | 15 |  | 49 | 154 | 87 | 15 |  | 34 | 68 | 114 | 14 |
| Herbaceous | *Rubia cordifolia* | Rubiaceae | - | - | - | - |  | - | 4 | - | - |  | - | 4 | - | - |
| Herbaceous | *Galium aparine* | Rubiaceae | 5 | 2 | - | - |  | 45 | 4 | 2 | 3 |  | 6 | - | - | - |
| Herbaceous | *Oxalis corniculata* | Oxalidaceae | 111 | 53 | - | - |  | 291 | 153 | 6 | - |  | 206 | 32 | - | - |
| Ferns | *Dryopteris crassirhizoma* | Dryopteridaceae | - | 1 | - | - |  | 2 | 3 | 1 | - |  | 3 | 1 | - | - |
| Ferns | *Athyrium brevifrons* | Athyriaceae | - | - | - | - |  | 46 | 33 | 1 | - |  | 15 | 17 | 1 | - |

**Appendix S2**

| Functional  groups | Species | Family | Spring | | | |  | Summer | | | |  | Autumn | | | |
| --- | --- | --- | --- | --- | --- | --- | --- | --- | --- | --- | --- | --- | --- | --- | --- | --- |
|  |  |  | N1 | N2 | N3 | N4 |  | N1 | N2 | N3 | N4 |  | N1 | N2 | N3 | N4 |
| Tree seedlings | *Acer tegmentosum* | Aceraceae | 17.0 | - | - | - |  | 7.4 | 6.9 | 4.8 | 3.1 |  | 12.6 | - | - | 3.1 |
| Tree seedlings | *Fraxinus mandshurica* | Oleaceae | 4.3 | - | - | - |  | 7.6 | 5.8 | - | 2.6 |  | 10.8 | 15.1 | - | - |
| Tree seedlings | *Ulmus japonica* | Ulmaceae | 5.2 | - | - | - |  | 8.2 | - | - | - |  | - | - | - | - |
| Tree seedlings | *Ulmus laciniata* | Ulmaceae | 3.6 | 22.5 | - | - |  | 13.0 | 22.1 | - | 23.5 |  | 6.0 | 18.6 | - | - |
| Tree seedlings | *Corylus mandshurica* | Betulaceae | - | - | - | - |  | 32.4 | 19.3 | - | - |  | 25.1 | 24.3 | 6.1 | - |
| Tree seedlings | *Acer mono* | Aceraceae | - | - | - | - |  | 32.3 | - | - | - |  | 6.5 | - | - | 23.3 |
| Tree seedlings | *Abies nephrolepis* | Pinaceae | - | - | - | - |  | 2.3 | 2.4 | - | - |  | 2.8 | 2.8 | - | - |
| Tree seedlings | *Pinus koraiensis* | Pinaceae | - | - | - | - |  | 7.6 | 6.0 | 6.3 | - |  | 5.6 | 5.8 | 7.3 | - |
| Tree seedlings | *Acer ukurunduense* | Aceraceae | - | - | - | - |  | 8.6 | - | - | - |  | - | - | - | - |
| Tree seedlings | *Prunus padus* | Rosaceae | - | - | - | - |  | - | 7.0 | - | - |  | - | - | - | - |
| Shrubs | *Acanthopanax senticosus* | Araliaceae | 28.1 | 18.1 | 5.1 | 15.2 |  | 41.3 | 22.7 | - | 25.9 |  | 29.3 | 17.2 | 19.7 | 19.5 |
| Shrubs | *Lonicera chrysantha* | Caprifoliaceae | 20.8 | - | - | - |  | 22.0 | 7.8 | 9.2 | - |  | 15.8 | 6.1 | - | - |
| Shrubs | *Philadelphus schrenkii* | Hydrangeaceae | 62.0 | - | - | - |  | 45.1 | 36.7 | 58.5 | 25.9 |  | 56.2 | - | - | - |
| Shrubs | *Euonymus pauciflorus* | Celastraceae | 37.0 | 10.6 | 20.1 | 6.6 |  | 29.5 | 6.5 | 8.2 | 3.2 |  | 18.7 | 10.4 | 5.8 | 5.0 |
| Shrubs | *Deutzia glabrata* | Hydrangeaceae | 46.3 | 41.0 | 23.5 | 59.2 |  | 34.3 | 10.8 | 25.6 | 29.0 |  | 31.1 | 21.9 | - | 11.2 |
| Shrubs | *Sorbaria sorbifolia* | Rosaceae | 39.9 | - | 13.6 | - |  | - | - | - | - |  | - | - | - | - |
| Shrubs | *Ribes mandshuricum* | Grossulariaceae | - | - | 20.4 | - |  | 16.4 | - | 25.8 | - |  | 17.1 | - | 37.0 | - |
| Shrubs | *Spiraea salicifolia* | Rosaceae | - | - | 41.1 | - |  | - | - | 25.7 | - |  | - | - | 49.8 | - |
| Shrubs | *Syringa reticulata* var.*amurensis* | Oleaceae | - | - | - | - |  | 30.6 | 63.9 | - | - |  | 25.1 | 80.0 | - | - |
| Shrubs | *Actinidia kolomikta* | Actinidiaceae | - | - | - | - |  | 31.2 | - | 33.7 | - |  | - | - | - | - |
| Shrubs | *Berberis poiretii* | Berberidaceae | - | - | - | - |  | 11.4 | - | - | - |  | - | - | - | - |
| Shrubs | *Vitis amurensis* | Vitaceae | - | - | - | - |  | - | 19.4 | - | - |  | - | 24.7 | - | - |
| Herbaceous | *Cardamine macrophylla* | Cruciferae | 7.5 | 7.9 |  | - |  | 8.7 | 14.1 | 25.9 | 11.5 |  | 6.9 | 8.4 | 10.0 | 7.2 |
| Herbaceous | *Lamium barbatum* | Labiatae | 15.3 | 8.1 | - | 18.3 |  | 22.3 | 16.4 | 16.1 | - |  | - | - | - | - |
| Herbaceous | *Paris verticillata* | Liliaceae | 13.6 | 16.3 | 4.7 | 19.2 |  | 6.2 | 3.5 | - | - |  | - | - | - | - |
| Herbaceous | *Carex pilosa* | Cyperaceae | 13.4 | 13.5 | 13.8 | 11.8 |  | 11.5 | 12.5 | 11.4 | 13.6 |  | 11.3 | 9.3 | 14.7 | 8.5 |
| Herbaceous | *Dichocarpum sutchuenense* | Ranunculaceae | 24.4 | 24.5 | 19.8 | 26.1 |  | 12.9 | 18.2 | 24.8 | 19.5 |  | - | - | 9.0 | - |
| Herbaceous | *Bupleurum sibiricum* | Umbelliferae | - | - | - | - |  | 2.0 | - | - | - |  | 10.5 | - | - | - |
| Herbaceous | *Heracleum hemsleyanum* | Umbelliferae | - | - | - | - |  | - | - | - | 96.5 |  | - | - | - | - |
| Herbaceous | *Parasenecio auriculatus* | Compositae | 9.6 | 9.9 | 8.0 | - |  | 10.4 | 6.4 | 13.1 | - |  | 15.9 | - | - | - |
| Herbaceous | *Adenocaulon himalaicum* | Compositae | - | - | - | - |  | - | - | - | - |  | - | - | - | 9.2 |
| Herbaceous | *Hylomecon japonica* | Papaveraceae | 19.4 | 17.1 | 14.1 | 12.7 |  | 7.6 | 9.5 | 14.3 | 13.9 |  | - | - | - | - |
| Herbaceous | *Polemonium caeruleum* | Polemoniaceae | - | - | 27.1 | - |  | 13.9 | - | - | - |  | - | - | - | - |
| Herbaceous | *Scutellaria pekinensis* | Labiatae | - | - | - | - |  | 3.5 | 4.7 | 22.0 | - |  | 4.2 | 5.2 | - | - |
| Herbaceous | *Theligonum macranthum* | Theligonaceae | 7.6 | 9.4 | 8.9 | 7.3 |  | 5.6 | 3.1 | 5.3 | 2.5 |  | 4.8 | - | - | - |
| Herbaceous | *Aruncus sylvester* | Rosaceae | - | - | - | 21.9 |  | - | - | - | - |  | - | - | - | - |
| Herbaceous | *Chrysosplenium pseudofauriei* | Saxifragaceae | 5.7 | 12.9 | 5.7 | 7.4 |  | 2.8 | 3.5 | 5.2 | 0.8 |  | 1.5 | 1.5 | 1.5 | - |
| Herbaceous | *Carex siderosticta* | Cyperaceae | - | - | - | - |  | 11.4 | - | 14.6 | - |  | - | - | - | 8.8 |
| Herbaceous | *Urtica laetevirens* | Urticaceae | - | - | 8.1 | - |  | 14.6 | 23.2 | - | - |  | 9.0 | 23.7 | - | - |
| Herbaceous | *Heracleum barbatum* | Umbelliferae | 20.6 | 12.3 | 24.2 | 12.9 |  | 39.2 | - | 29.9 | - |  | - | - | - | - |
| Herbaceous | *Veratrum dahuricum* | Liliaceae | - | - | 21.3 | - |  | - | - | 13.1 | - |  | - | - | - | - |
| Herbaceous | *Rubia sylvatica* | Rubiaceae | 7.9 | - | 4.6 | 4.7 |  | 17.5 | 1.3 | 2.2 | 0.9 |  | - | - | - | - |
| Herbaceous | *Diarrhena manshurica* | Gramineae | - | - | - | - |  | 21.6 | - | - | 17.6 |  | - | - | - | 9.7 |
| Herbaceous | *Caltha palustris* | Ranunculaceae | 19.5 | - | 25.4 | 19.3 |  | - | 20.3 | 27.6 | - |  | - | 12.3 | 4.4 | - |
| Herbaceous | *Equisetum hyemale* | Equisetaceae | 30.2 | - | - | - |  | 31.6 | - | - | - |  | 32.8 | - | 40.1 | - |
| Herbaceous | *Viola collina* | Violaceae | - | - | - | - |  | 3.8 | 1.1 | - | - |  | 2.7 | 2.1 | - | 2.2 |
| Herbaceous | *Brachybotrys paridiformis* | Boraginaceae | 5.0 | - | - | - |  | - | - | 15.6 | - |  | - | - | - | - |
| Herbaceous | *Circaea alpina* | Onagraceae | - | - | - | - |  | - | 42.1 | 35.7 | - |  | - | - | - | - |
| Herbaceous | *Impatiens nolitangere* | Balsaminaceae | 2.9 | 6.8 | 3.3 | 7.4 |  | 15.0 | 23.2 | 36.9 | 33.6 |  | - | 32.6 | - | - |
| Herbaceous | *Thalictrum przewalskii* | Ranunculaceae | - | - | - | - |  | - | - | 12.9 | - |  | - | - | - | - |
| Herbaceous | *Phryma leptostachya* subsp*. asiatica* | Labiatae | - | - | - | - |  | 17.6 | - | - | - |  | 24.3 | 38.2 | - | - |
| Herbaceous | *Pilea pumila* | Urticaceae | - | - | - | - |  | - | - | - | - |  | - | 7.8 | - | - |
| Herbaceous | *Eranthis stellata* | Ranunculaceae | 6.7 | 5.1 | 7.6 | 5.9 |  | - | - | - | - |  | - | - | - | - |
| Herbaceous | *Carex ussuriensis* | Cyperaceae | - | - | 11.4 | 8.7 |  | 20.9 | - | 18.2 | - |  | - | - | - | - |
| Herbaceous | *Aconitum volubile* | Ranunculaceae | 25.1 | 12.7 | 28.9 | 22.8 |  | - | 18.0 | 35.9 | 51.6 |  | - | - | - | - |
| Herbaceous | *Adoxa moschatellina* | Adoxaceae | 6.1 | 6.6 | 7.3 | 6.6 |  | - | 2.6 | 2.6 | - |  | - | - | - | - |
| Herbaceous | *Maianthemum bifolium* | Liliaceae | 6.7 | 10.2 | 6.1 | 10.2 |  | 5.7 | 7.4 | 7.1 | 13.0 |  | 3.5 | - | - | - |
| Herbaceous | *Urtica angustifolia* | Urticaceae | 7.9 | 6.0 | - | - |  | 29.6 | 22.2 | 39.5 | - |  | - | 14.7 | 9.5 | - |
| Herbaceous | *Aegopodium alpestre* | Umbelliferae | 5.7 | 7.4 | 5.5 | 6.5 |  | 4.8 | 4.8 | 6.0 | 5.9 |  | 5.2 | 4.5 | 4.5 | 4.1 |
| Herbaceous | *Corydalis yanhusuo* | Fumariaceae | 7.3 | 7.4 | 10.1 | 8.9 |  | - | - | - | - |  | - | - | - | - |
| Herbaceous | *Saussurea amurensis* | Compositae | 6.5 | - | 8.9 | 24.8 |  | - | - | 31.8 | 60.7 |  | - | - | - | 43.2 |
| Herbaceous | *Carex rigescens* | Cyperaceae | 15.6 | - | 7.8 | 14.1 |  | 9.9 | 8.6 | 7.4 | 2.8 |  | 7.1 | 7.3 | 11.8 | 4.7 |
| Herbaceous | *Anemone cathayensis* | Ranunculaceae | 8.3 | 10.9 | 7.5 | 9.2 |  | - | - | - | - |  | - | - | - | - |
| Herbaceous | *Filipendula palmata* var. *glabra* | Rosaceae | 10.5 | 12.3 | 10.3 | 18.6 |  | 19.7 | 23.1 | 17.4 | 16.0 |  | 11.9 | 13.3 | 8.3 | 6.2 |
| Herbaceous | *Rubia cordifolia* | Rubiaceae | - | - | - | - |  | - | 10.3 | - | - |  | - | 5.1 | - | - |
| Herbaceous | *Galium aparine* | Rubiaceae | 4.8 | 9.6 | - | - |  | 4.0 | 3.6 | 5.2 | 3.0 |  | 6.6 | - | - | - |
| Herbaceous | *Oxalis corniculata* | Oxalidaceae | 3.9 | 3.6 | - | - |  | 3.4 | 3.0 | 2.7 | - |  | 1.6 | 1.3 | - | - |
| Ferns | *Dryopteris crassirhizoma* | Dryopteridaceae | - | 17.5 | - | - |  | 31.0 | 26.5 | 22.1 | - |  | 37.4 | 40.1 | - | - |
| Ferns | *Athyrium brevifrons* | Athyriaceae | - | - | - | - |  | 16.5 | 13.8 | 10.3 | - |  | 12.3 | 13.2 | 18.6 | - |
